# Particles in Practice: How Ultrafines Disseminate in the Body

**Published:** 2005-11

**Authors:** Bob Weinhold

Ultrafine particles (UFPs), those less than 100 nanometers in diameter, have existed for millennia in natural settings. But with the significant increase in UFPs resulting from human activities in the past few centuries (largely through combustion processes) and the potential for a deluge of nanoparticles as that industry gears up, are ancient human bodily defenses up to the substantial new hazards they now face? Findings by a team of Swiss, German, and Canadian researchers suggest that animals may be largely defenseless against the rapid dissemination of UFPs into cells throughout the body **[*EHP* 113:1555–1560]**. Their findings, which include the first evidence of how individual particles are distributed within the lung, raise some concerns, especially since UFPs often end up in locations within cells where the tiny particles can impair many cellular functions.

General knowledge about the rapid penetration of UFPs into various body organs has surfaced in the past few years, but the specific distribution and mechanisms remain largely unknown. To explore the distribution, the research team performed two parallel sets of experiments.

In the first set of experiments, they investigated the spread of titanium dioxide UFPs in rats after a 1-hour inhalation of an aerosol containing the material. The team then evaluated lung tissue taken from the rats either 1 or 24 hours after inhalation.

They found that on average, 24% of the inhaled titanium dioxide they detected had penetrated cells throughout the lung and the bloodstream just 1 hour after inhalation. Within cells in different lung compartments, there was no difference in the 1-hour and 24-hour samples, suggesting that UFPs can easily move between compartments. The team continues to investigate what happens with the remaining 76% of the particles and with those that enter the bloodstream. There is evidence the particles spread throughout the body.

Of the particles they did find, 79.3% lodged in cells on the inner surface of airways and alveoli, 11.3% were within capillaries, 4.8% were within connective tissue, and 4.6% were within epithelial or endothelial cells. The researchers were surprised to find that most of the particles in the cellular cytoplasm were not attached to the membrane, as would have been expected if the particles had been encapsulated through endocytosis or phagocytosis. Floating in the cytoplasm, the particles can access many of the structures within the cell, such as the nucleus and mitochondria, increasing the potential toxicity of the particles.

In the second set of experiments, the researchers explored the movement of three sizes of fluorescent polystyrene UFPs and of gold UFPs after the particles were introduced to cultures of swine macrophages and human red blood cells. They found all three particle sizes (1.0, 0.2, and 0.078 micrometer) penetrated the swine macrophages, though in perplexingly different proportions—only 21% of the macrophages contained the medium size, while 77% contained the smallest and 56% contained the largest. In human red blood cells, they found the smallest and medium sizes, but not the largest.

The experiments did not offer evidence about exactly how the tiny, insoluble particles disseminate so extensively and rapidly into so many different cells, but the researchers note that other experiments have demonstrated a number of possible mechanisms. The researchers also note their findings are specific to just the few substances they studied, and differ in some ways from those for iridium, one of the few other materials evaluated in some detail.

## Figures and Tables

**Figure:**
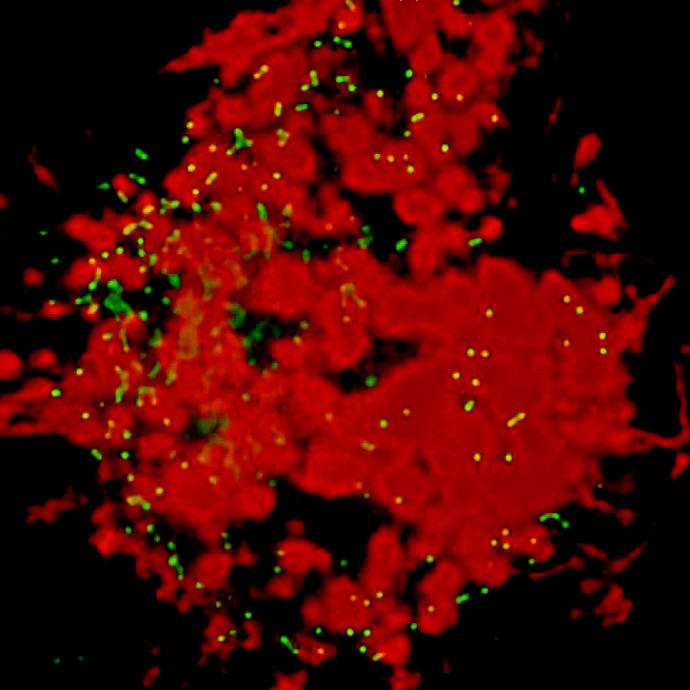
Ultrafine infusion. A series of recent experiments demonstrates that ultrafine particles are widely disseminated in a variety of cells. A micrograph of one such experiment shows 0.2-micrometer fluorescent polystyrene particles (green) taken up by a macrophage (red).

